# Association of rs5051 and rs699 polymorphisms in angiotensinogen with coronary artery disease in Iranian population: A case-control study

**DOI:** 10.1097/MD.0000000000037045

**Published:** 2024-03-15

**Authors:** Maryam Mirahmadi, Aref Salehi, Masoud Golalipour, Azam Bakhshandeh, Majid Shahbazi

**Affiliations:** a Medical Cellular and Molecular Research Center, Golestan University of Medical Sciences, Gorgan, Iran; b Department of Medical Genetics, Faculty of Medicine, Tarbiat Modares University, Tehran, Iran; c Department of Exomine, PardisGene company, Tehran, Iran; d Ischemic Disorders Research Center, Golestan University of Medical Sciences, Gorgan, Iran; e AryaTinaGene, Biopharmaceutical Company, Gorgan, Iran.

**Keywords:** A-6G, association, M235T, polymorphism, renin-angiotensin-aldosterone system

## Abstract

Coronary artery disease (CAD) is the third most common cause of mortality globally (with 17.8 million deaths annually). Angiotensinogen (AGT) and polymorphisms in this gene can be considered as susceptibility factors for CAD. We performed a retrospective case-control study to determine the correlation of AGT rs5051 and rs699 polymorphisms with CAD in an Iranian population. We genotyped 310 CAD patients and 310 healthy subjects using polymerase chain reaction-based methods. To confirm the accuracy of the screening approach, 10% of genotyped subjects were validated using gold-standard Sanger Sequencing. To evaluate the effect of the candidate polymorphisms, white blood cells were randomly purified from the subjects and AGT expression was measured by quantitative reverse transcriptase-polymerase chain reaction. Sex stratification indicated a significant correlation between CAD and male sex (*P* = .0101). We found a significant association between the rs5051 A allele (*P *= .002) and the rs699 C allele, and CAD (*P* = .0122) in recessive and dominant models. Moreover, our findings showed a significant association of the haplotype, including the rs5051 A/A and rs699 T/C genotypes, with CAD (*P* = .0405). Finally, AGT mRNA levels were significantly decreased in patients harboring the candidate polymorphisms (*P* = .03). According to our findings The AGT rs5051 A and AGT rs699 C alleles are predisposing variants of CAD risk and severity in the Iranian population.

## 1. Introduction

Coronary artery disease (CAD) ranks third in terms of mortality and disability and accounts for 17.8 million deaths annually.^[1,2]^ The combination of several genes and environmental risk factors has led to the classification of CAD as a complex disease. High blood pressure, smoking, obesity, and diabetes mellitus are the common risk factors for CAD. Family history is also associated with the risk of mortality of CAD.^[3]^

The renin-angiotensin-aldosterone system (RAAS) is a key modulator of cardiovascular and renal function. Clinical trials have demonstrated that inhibiting this system would reduce the risk of coronary events.^[4,5]^ RAAS is involved in many diseases, including atrial fibrillation, congestive heart failure, myocardial fibrosis, cardiomyopathy, hypertension, and type 2 diabetes mellitus.^[6–10]^ Angiotensinogen (AGT) plays a crucial role in RAAS. Angiotensin-converting enzyme converts Angiotensin I to Angiotensin II, which acts as a vasoconstrictor and a growth-promoting factor in the cardiovascular system. Angiotensin II is responsible for fibroblast proliferation and plaque formation.^[11]^

Single-nucleotide polymorphisms (SNP) can alter the expression and function of host proteins. rs5051 (A-6G), which is located in the promoter region, and rs699 (M235T), a missense variant replacing methionine with threonine in exon 2, are two SNPs that may affect the expression or functionality of AGT.^[12–14]^ It has been reported that these two SNPs are tightly linked, and according to dbSNP, the rs5051 A and rs699 T alleles are ancestral alleles. Previously, we showed a correlation between polymorphisms in PDGF-B, IL-10, IL-18, IFN-γ, and CAD.^[15–18]^

As AGT is an important regulator of cell proliferation and vessel thickness, we conducted a retrospective case-control study to determine the correlation between rs5051 and rs699 polymorphisms in this gene and CAD. Using mutagenically separated- polymerase chain reaction (MS-PCR) and sequence-specific primer-PCR (SSP-PCR), our findings indicated that there is a significant association between A allele of rs5051 and C allele of rs699 polymorphisms and CAD.

## 2. Material and methods

### 2.1. Sample collection

To evaluate the impact of genetic variation on susceptibility to CAD, we randomly screened 310 patients and 316 control individuals (mean age, 30–60 years) obtained from the Kouwsar Heart Center of Golestan University of Medical Sciences, Gorgan, Iran. The local Ethical Committee of Golestan University of Medical Sciences authorized the study protocol and provided written informed consent. The cardiologist-approved CAD diagnosis was based on the clinical and paraclinical findings. The inclusion criterion for invasive angiography was 70% stenosis in at least one major coronary artery in the heart; the control subjects were healthy individuals with normal angiography findings (15% stenosis). Moreover, the control group lacked symptoms of myocardial ischemia during exercise and had normal electrocardiograms at rest.^[19]^

### 2.2. DNA extraction and genotyping

Genomic DNA (gDNA) was extracted from 10 mL of peripheral blood samples using a modified phenol–chloroform extraction method, and samples were stored at −20°C until analysis^[20]^ Genotyping of rs5051 and rs699 polymorphisms was performed using MS-PCR and SSP-PCR^[21]^ The human growth hormone gene was used as an internal control for the rs699 polymorphism PCR analysis. The primer sequences and product sizes for each primer set are listed in Table 1.

**Table 1:** Primers used in this study.

Amplified factor	Direction	Sequence (5’ to 3’)	Size (bp)	Gene accession number
SSP-PCR				
AGT rs699	Forward-C	TGTCCACACTGGCTCCCG	168	NG_008836.1
	Forward-T	TGTCCACACTGGCTCCCA		
	Reverse	ACCTGAAGCAGCCGTTTGT		
hGH-N	Forward	GCCTTCCCAACCATTCCCTTA	429	NG_011676.1
	Reverse	TCACGGATTTCTGTTGTG		
MS-PCR				
AGT rs5051	Forward	GTGTCGCTTCTGGCATCTGTCCTTCTGG		NG_008836.1
	Reverse-A	TACCCAGAACAACGGCAGCTTCTTCCACT	170	
	Reverse-G	CCGGTTACCTTCTGCTGTACAGCCCAGAACAACGGCAGCTTCTTCCATC	192	
qRT-PCR				
PGK1	Forward	GCAGATTGTGTGGAATGGTC	101	NM_000291.3
	Reverse	CCCTAGAAGTGGCTTTCACC		
AGT	Forward	ACAATGAGAGTACCTGTGAGCA	90	NG_008836.1
	Reverse	TCTTGGCCTGAATTGGAGCAG		

bp = base pair.

To detect the rs5051 polymorphism, a PCR reaction was conducted in a 25 µL reaction solution containing 100 ng gDNA, 25 mM MgCl2, 10 mM of each dNTP, one unit Taq DNA polymerase (Thermo Fisher, United States), and 100 pmol of each primer (Nucleics, Germany). PCR conditions were as follows: initial denaturation at 94°C for 5 min followed by 35 cycles of denaturation at 94°C for 30 seconds, annealing at 59°C for 30 seconds, and a final extension at 72°C for 5 minutes. PCR for the detection of the rs699 polymorphism was conducted in a 15 µL reaction solution containing 100 ng gDNA, 13 μl master mix containing 25 mM MgCl2, 10 mM of each dNTP, one unit Taq DNA polymerase (Thermo Fisher, United States), 12% sucrose (Merck, Germany) and 5 pmol of each specific primer (Nucleics, Germany). The SSP-PCR reaction was performed using the following program: initial denaturation at 95°C for 1 minute, followed by 10 cycles of 15 seconds at 95°C, 50 seconds at 65°C, 40 seconds at 72°C, followed by 20 cycles of 20 seconds at 95°C, 50 seconds at 57°C, and 50 seconds at 72°C, and 5 min at 72°C as final extension. The PCR products of the SSP-PCR and MS-PCR experiments were electrophoresed on agarose gel (Merck, Germany) and visualized using a gel documentation system (UVITEC, UK).

### 2.3. Quantitative reverse transcriptase PCR

Total RNA was isolated from cultured leukocytes in peripheral blood using the TRIzol reagent (Invitrogen, Karlsruhe, Germany). The quality of the extracted RNA was determined by electrophoresis on a 1% agarose gel and quantified using NanoDrop ND 1000 (Thermo Fisher, United States). Complementary DNA (cDNA) was synthesized using 1 µg of total RNA, which was reverse-transcribed using oligo dT and the Revert Aid First Strand cDNA Synthesis Kit (Fermentas, Sankt Leon-Rot, Germany). The cDNA samples were stored at −80°C until being analyzed using Real-Time PCR System 7300 (Applied Biosystems, USA). Relative gene expression levels of AGT were normalized to Phosphoglycerate Kinase 1 (PGK1) in triplicate using Maxima SYBR Green/Rox QPCR Master Mix (2X) (Thermo Fisher, United States), according to the manufacturer instructions. The mRNA levels were calculated using the 2^−ΔΔCt^ method.^[22]^

### 2.4. Statistical analysis

All data were analyzed using GraphPad software v.8 (San Diego, CA). Fisher exact test was used to check for deviation from the Hardy - Weinberg equilibrium for each polymorphism in patients and controls separately. Statistical significance was set at *P* < .05. The χ^2^-test was used to compare allele and genotype frequencies between the CAD cases and the healthy control group, and to analyze the potential differences in genotype frequency between the patient groups with a number of stenosis vessels. Data are presented as mean ± SD for parametric variables and percentages for non-parametric variables.

## 3. Results

The genotype frequencies of rs5051 in cases (χ^2^ = 0.48, *P* = .78) and controls (χ^2^ = 0.4, *P* = .81), and the genotype frequencies of rs699 in cases (χ^2^ = 3.01, *P* = .22) and controls (χ^2^ = 1.3, *P* = .52), were consistent with Hardy-Weinberg law. Moreover, sex and ethnic group stratification showed a significant association between male sex and CAD (*P* = .0101); however, there was no association between ethnic subgroups and CAD (Table 2).

**Table 2:** The association analysis of ethnic subgroups and gender status with CAD disease.

Variables Ethnic group	Control, n (%)	Case, n (%)	OR (95% CI)	*P* value
Fars	228 (73.5)	235 (75.8)	Ref	-
Turkman	47 (15.2)	40 (12.9)	0.8 (0.5–1.3)	.4834
Sistani	35 (11.3)	35 (11.3)	1 (0.6–1.6)	1
Female	167 (53.9)	134 (43.2)	Ref	-
Male	143 (46.1)	176 (56.8)	1.5 (1.1–2.1)	**.0101**

Comparison analysis was done by χ^2^-test. Data are presented as number (%).

CI = confidence interval, n = number, OR = odds ratio, Ref = reference.

Complementary data, including the allele and genotype frequencies of the rs5051 and rs699 polymorphisms in the case and control groups, are shown in Table 3.

**Table 3:** The allelic and genotypic distribution of AGT rs5051 and rs699 polymorphisms in the two groups.

Variable	Control, n (%)	Case, n (%)	OR (95% CI)	*P v*alue
AGT rs5051				
Alleles				
G	304 (49)	249 (40.2)	Ref	-
A	316 (51)	371 (59.8)	1.4 (1.1–1.8)	**.0020**
Genotypes				
G/G	75 (24.2)	50 (16.1)	Ref	-
G/A	154 (49.7)	149 (48.1)	1.4 (0.9–2.2)	.0890
A/A	81 (26.1)	111 (35.8)	2 (1.3–3.3)	**.0027**
Model of inheritance				
Recessive (A/A vs A/G + G/G)			1.6 (1.1–2.2)	**.0117**
Dominant (A/A + G/A vs A/A)			1.6 (1.1–2.5)	**.0161**
Overdominant (G/A vs G/G + A/A)			0.94 (0.7–1.3)	.7480
AGT rs699				
Alleles				
T	312 (50.3)	267 (43.1)	Ref	-
C	308 (49.7)	353 (56.9)	1.3 (1–1.7)	**.0122**
Genotypes				
T/T	72 (22.6)	50 (16.1)	Ref	-
T/C	168 (54.2)	167 (53.9)	1.4 (0.9–2.2)	.1121
C/C	70 (23.2)	93 (30)	1.9 (1.1–3.2)	**.0085**
Model of inheritance				
Recessive (C/C vs T/C + T/T)			1.5 (1–2.1)	**.0446**
Dominant (C/C + T/C vs T/T)			1.6 (1–2.4)	**.0336**
Overdominant (T/C vs T/T + C/C)			1 (0.7–1.4)	1

Our statistical analyses showed a significant association between CAD and rs5051 A allele frequency (*P* = .002) and A/A genotype (*P* = .0027). Furthermore, the distribution of the frequencies of the rs699 C/C genotype (*P* = .0085) and the C allele (*P* = .0122) showed statistically significant differences between the case and control groups. Three inheritance models (recessive, dominant, and overdominant) were considered for the two SNPs, and a significant association between re5051 and rs699 was detected in the recessive and dominant inheritance models (*P* < .05) (Table 3).

Furthermore, our results showed an increased susceptibility to CAD in that the genotype combinations of rs5051 A/A and rs699 T/C (*P* = .0405) (Table 4).

**Table 4:** Haplotype frequencies of AGT polymorphisms in the two groups.

Genotype combination		Haplotype	Control (n)	Case (n)	OR (95% CI)	*P v*alue
rs5051	(rs699)					
G/G	T/T	H1	15	11	Ref	-
	T/C	H2	41	23	0.8 (0.3–2.2)	.6349
	C/C	H3	19	16	1.1 (0.4–3.6)	1
G/A	T/T	H4	34	22	0.9 (0.3–2.5)	.8131
	T/C	H5	95	82	1.2 (0.5–3)	.8337
	C/C	H6	25	45	2.4 (0.9–6.8)	.0644
A/A	T/T	H7	23	17	1 (0.3–3.1)	1
	T/C	H8	32	62	2.6 (1–7.1)	**.0405**
	C/C	H9	26	32	1.7 (0.6–4.8)	.3470

H = Haplotype.

The distribution of genotype frequencies in the case group according to the number of affected arterial vessels is presented in Table 5.

**Table 5:** Genotype frequencies of rs5051 and rs699 polymorphisms in the CAD patients according to the number of affected arteries.

Genotype	Control (n)	1-VD (n)	*P v*alue	2-VD (n)	*P v*alue	3-VD (n)	*P v*alue
rs5051							
G/G	75	23	Ref	14	Ref	13	Ref
A/G	154	56	.5783	49	.1234	44	.1534
A/A	81	36	.2831	25	.2094	50	**.0002**
rs699							
T/T	72	21	Ref	14	Ref	15	Ref
C/T	168	69	.2723	43	.5162	55	.1763
C/C	70	32	.1983	21	.2656	40	**.0038**

Our results indicated that the A/A genotype of rs5051 was significantly associated with three-vessel disease (3-VD) (*P* < .001). Moreover, a significant association between this disease and the C/C genotype of rs699 (*P* = .0038) was also observed.

**Figure 1.**
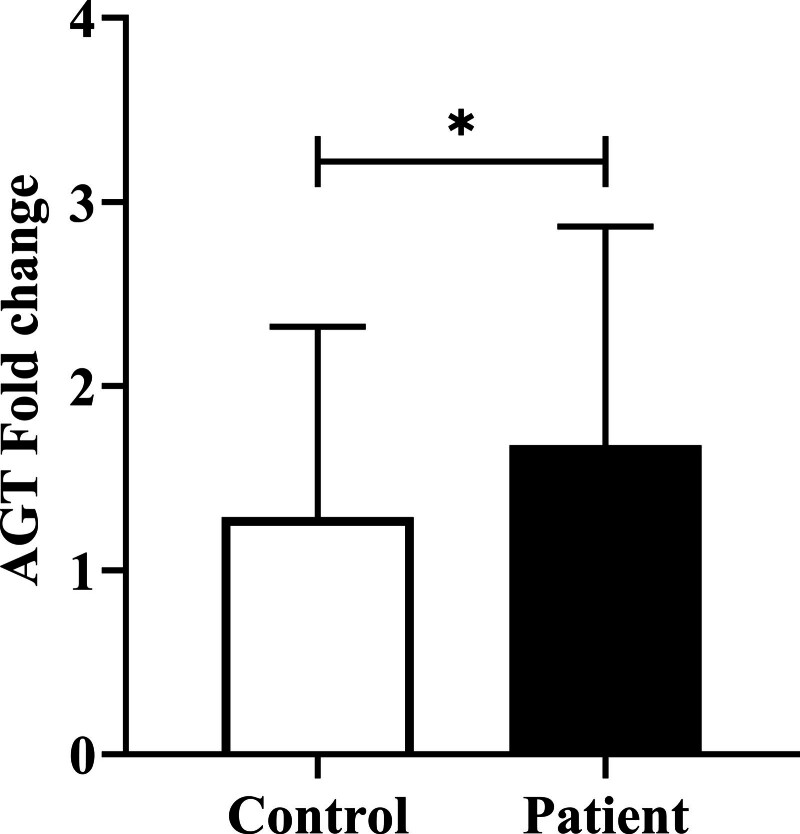
AGT mRNA level was quantified in random cases and control subjects by qRT-PCR. AGT expression was normalized to that of PGK1. The AGT expression level in CAD patients was significantly increased compared to that in the controls.

To assess the effect of the two risk factor SNPs, rs5051 and rs699, on the expression of the AGT gene in case and the control groups, we randomly selected 80 patients harboring risk alleles and 80 control subjects of the same age or peripheral conditions such as smoking. Our results indicated that AGT expression was significantly higher in patients with CAD than in the controls (*P* = .03) (Figure 1).

## 4. Discussion

As a multifactorial disease, CAD is one of the most critical concerns regarding population health worldwide and is associated with multiple environmental and genetic factors.^[23]^ Previously, heterogeneity in CAD severity in the Iranian population was associated with an increased risk in Gilak and Turks.^[24,25]^ Here, we compared the frequency of CAD in the Iranian North population, which includes three major groups: Fars, Turkmen, and Sistani. Our observations did not indicate a significant association between CAD and ethnicity. It is primarily considered that CAD is mainly observed in men.^[26]^ In this study, sex stratification indicated that CAD occurred more frequently in men than in women. AGT is a major effector of the RAAS system that activates the mitogen-activated protein pathway and may have toxic effects on myocardial cells.^[27]^ In addition, increased plasma renin activity is associated with mortality in CAD patients.^[28]^ AGT polymorphisms are likely to play a role in CAD pathogenesis by increasing plasma angiotensin levels.^[29,30]^ Two SNPs, rs5051 and rs699, are in tight linkage disequilibrium.^[30]^

We observed a significant association between the rs5051 A allele and rs699 C allele with CAD. Our findings correlate with those of several prior studies, in which AGT polymorphisms were associated with susceptibility to higher plasma angiotensin levels.^[31,32]^ Jia et al (2014) reported a significant association between rs5051 and AGT levels in Chinese CAD patients.^[28]^ However, several studies have reported conflicting results, with no significant association observed between rs699 and CAD.^[33,34]^ Our findings indicate that the A/A genotype of rs5051 and the CC genotype of rs699 were significantly correlated with CAD. Previously, it was indicated that these two genotypes were correlated with increased angiotensin II levels.^[28,35]^ The A allele of rs5051 and C allele of rs699 are the original forms of the gene and are associated with higher angiotensin II levels. In addition, rs5051 G and rs699 T alleles are neomorphs associated with lower angiotensin II levels.^[35]^ In this study, we observed that rs5051 and rs699 were both inherited in recessive and dominant models. Khatami et al (2017) reported that the Iranian population rs699 C allele is significantly associated with CAD and is inherited in both dominant and overdominant models. Our findings in this study confirmed an association between the rs699 C allele and CAD. However, our statistical analysis did not show any significant association between this SNP and overdominant model, probably because of differences in ethnic groups. Our ethnic groups were categorized into three subgroups: Fars, Turkman, and Sistani, mostly restricted to the Iranian North population. However, Khatami et al did not identify the ethnic groups in their study.^[36]^ A strong association between male sex and CAD susceptibility was observed (*P* = .01). It appears that the gender gap in CAD occurs because encounters with stressful events may be less physiologically, behaviorally, and emotionally adaptive.^[37]^

We used haplotype structure to appropriately characterize the common variation, because it is more valuable than SNP analysis. We assessed the combinations of these two SNPs. In our study, the most common haplotype was H5. H8, including the rs5051 AA and rs699 TC genotypes, was significantly associated with CAD. This finding correlates with those of previous multivariate studies in the Taiwanese population. The haplotypes consisted of AACC, emphasizing their susceptibility association with CAD.^[38]^

Moreover, in another multivariant case-control study in the eastern Indian population, the haplotype including the rs5051 A and rs699 C alleles was introduced as a predisposing factor.^[39]^ Although the polygenic effect has barely been addressed in genetic studies of CAD, most have reported a synergistic contribution of AGT rs699 MM and Angiotensin-converting enzyme I/D DD genotypes with the development of CAD.^[40]^ This indicates that the A allele of the rs5051 polymorphism performs specific interactions between at least one transcription factor and the AGT promoter, leading to increased expression of AGT.

This study also analyzed the impact of AGT polymorphisms rs5051 and rs699 on the severity of coronary lesions in patients with CAD. Our findings indicate that the rs5051 AA and rs699 CC genotypes were significantly associated with the most severe coronary lesions (3-VD). Further evidence of the high expression level of AGT was provided by the elevated angiotensin II expression level, which appeared in athetotic changes in other tissues such as the kidneys.^[41]^ Since rs5051 is in very tight linkage disequilibrium with rs699, we examined the AGT gene expression levels in the case and control groups. The association of the rs5051 A allele with disease and high expression levels of the AGT gene in patients in our study is supported by the observation of a significant association between the rs5051 AA genotype and rs699 CC genotype and 3-VD.

## 5. Conclusion

In conclusion, we identified a significant association between the rs5051 A and rs699 C alleles and CAD in recessive and dominant models. Therefore, these two polymorphisms can be considered as predisposing diagnostic markers in Iranian patients with CAD.

## Author contributions

**Conceptualization:** Maryam Mirahmadi, Majid Shahbazi.

**Data curation:** Masoud Golalipour, Majid Shahbazi.

**Formal analysis:** Maryam Mirahmadi.

**Funding acquisition:** Majid Shahbazi.

**Investigation:** Maryam Mirahmadi, Masoud Golalipour, Azam Bakhshandeh, Majid Shahbazi.

**Methodology:** Maryam Mirahmadi, Azam Bakhshandeh.

**Project administration:** Maryam Mirahmadi, Majid Shahbazi.

**Resources:** Aref Salehi, Majid Shahbazi.

**Software:** Maryam Mirahmadi.

**Supervision:** Majid Shahbazi.

**Validation:** Aref Salehi, Masoud Golalipour, Majid Shahbazi.

**Writing – original draft:** Maryam Mirahmadi.

**Writing – review & editing:** Masoud Golalipour, Majid Shahbazi.
